# Travis County Medical Society—“Germ Theory of Disease”

**Published:** 1886-07

**Authors:** 


Travis County Medical Society.—At the regular meeting of this staunch society, July 1st., there was an interesting discussion on a query propounded by a committee appointed to select a subject for discussion, "What has been your experience in treatment, medical and surgical, based on the germ theory of disease?" Dr. Burt read an elaborate paper—the last he ever wrote—in which he arrayed all the known facts against the acceptance of the germ theory. This was discussed by Drs. McLaughlin, Hadra, Swearingen, Morris, and others of the society, and by most of the visiting physicians present. The meeting was honored by the presence of Drs. Tyner and Terrell, of San Antonio, Gruber, of New Braunfels, Thorpe, of Liberty Hill, Christian and King, of Burnet, Hamilton and Cook, of Hornsby, and Dr. Florence E. Collins, of Austin, who was admitted to membership. After the business was finished, the brethren adjourned from "labor to refreshment," and discussed peach cream and caramels till a late hour.

We are pleased to say this society is in a flourishing condition. This departure, the inviting of visitors from neighboring cities, was instituted in the hope that others would follow the example, and in that way bring about a more intimate relation and acquaintance amongst the physicians of this State.

